# Diversifying selection identified in immune epitopes of bovine coronavirus isolates from Irish cattle

**DOI:** 10.1099/jgv.0.002019

**Published:** 2024-08-19

**Authors:** Tristan Russell, Jose Maria Lozano, Cailyn Challinor, Gerald Barry

**Affiliations:** 1 UCD School of Veterinary Medicine, University College Dublin, Dublin, Ireland; 2 Centre for Experimental Pathogen Host Research (CEPHR), University College Dublin, Dublin, Dublin 4, Ireland; 3 Department of Agriculture, Food and the Marine, Central Veterinary Research Laboratory, Celbridge, Ireland

**Keywords:** bovine coronavirus, immune epitopes, Ireland, phylogenetics, selective pressure

## Abstract

*Bovine betacoronavirus* (BoCoV) is a pneumoenteric pathogen of cattle that is closely related to human coronavirus OC43. Vaccines are administered to protect against diseases caused by BoCoV, but knowledge gaps exist with regard to correlates of protection and the effect of immune evasion on driving evolution. In this study, immune epitopes were mapped onto BoCoV structural proteins, including spike and haemagglutinin esterase (HE), and then supported with targeted gene sequencing of Irish clinical isolates and selective pressure analysis. Increased prevalence of diversifying selection and amino acid changes in some mapped immune epitopes suggests that immune escape is selecting for non-synonymous mutations arising in these regions. Selection analysis and sequencing provided increased support for neutralising antibody (nAb) epitopes compared to others, suggesting that nAbs are an important arm of the immune response to BoCoV. Phylogenetic analysis of spike and HE sequences showed that Irish isolates from this study were in the European clade, except for one HE sequence that sat in the Asian/American clade, while the spike gene of this sample was in the European clade. Recombination between a European and an Asian/American isolate would give rise to such a sequence. This study has gathered evidence suggesting that pressure to evade the nAb response is contributing to BoCoV evolution.

## Introduction

*Bovine betacoronavirus* (BoCoV) is a pneumoenteric pathogen of *Bos taurus*, although closely related viruses have been isolated from several other ruminant species and some non-ruminant mammals [1]. In cattle it causes winter dysentery and calf diarrhoea [2] and is associated with bovine respiratory disease [3]. An inactivated trivalent vaccine administered to heifers pre-calving is available to provide newborn calves with protection against the enteric form of BoCoV, but the frequency of its use in Ireland is not publicly available or unknown [4]. In Ireland a retrospective study published in 2014 found that of the five viruses included in the screen, BoCoV was the most frequently detected in calves with respiratory disease (22.9% of 1364) [5]. BoCoV is also frequently detected in pneumonia cases as part of co-infections [6].

As a member of the subgenus *Embecovirus*, BoCoV encodes five structural proteins, including four conserved across the family *Coronaviridae* – spike, nucleocapsid (N), membrane and small envelope – as well as a surface protein unique to the subgenus *Embecovirus* – haemagglutinin esterase (HE). HE is a receptor-degrading enzyme, which facilitates virus entry and release by targeting the 9-O-acetylated sialic acid residues BoCoV attaches to during entry [7]. Infection-induced humoral immune responses against spike and HE can protect cattle from disease [1,8, 9], while antibodies generated in response to vaccine can recognise antigens derived from clinical isolates [10]. However, calf anti-BoCoV antibody titres induced by vaccinations of dams with vaccines for enteric disease do not protect from respiratory disease [11,12].

Our current understanding of BoCoV immune epitopes, correlates of protection, and the effect of immune evasion on BoCoV evolution is limited [1]. To further understand which viral proteins are targeted by the immune system and to assess the contribution of immune evasion pressure on protein sequence variation, mapped immune epitopes of BoCoV structural proteins were combined with selective pressure analysis in this study. There are already full-genome sequences of 24 BoCoV isolated from cattle in 2019/20 from across Ireland available publicly [13], as well as 10 S1 domain sequences and 1 full-length spike sequence from isolates collected in the province of Munster in 2010/11 [4]. Here the spike and HE genes of 29 Irish clinical isolates were sequenced and compared with other BoCoV isolates obtained nationally and internationally. Phylogenetic analysis of the spike and HE sequences identified residues where amino acid changes have occurred compared to a commonly used vaccine strain. We present evidence of selective pressure and amino acid changes occurring on residues associated with mapped immune epitopes, which may lead to reduced efficacy of currently used vaccines.

## Methods

### Samples and detection of bovine coronavirus

Nasopharyngeal samples collected from 29 cattle displaying symptoms of respiratory or enteric illness during 2022/23 in Ireland were submitted to the Department of Agriculture, Food and the Marine’s Virus reference laboratory (Table 1). Samples were tested for the presence of respiratory pathogens by qPCR. Those positive for BoCoV were identified using a previously developed TaqMan qPCR protocol [14].

**Table 1.** Sample details and GenBank accession numbers. All isolates were collected from different farms except those marked with matching symbols (* and †)

Sample no.	GenBank accession	Location (county)	Date of collection	Pathology	Host
1	OR271239	Galway	30 March 2022	nd	na
2	OR271240	Waterford	11 May 2022	nd	na
3	OR271241	Sligo	27 April 2022	Dead, pneumonia, *Histophilus somni* and *Mannheimia haemolytica* co-infection	3 w
4*	OR271242	Laois	8 April 2022	nd	2 m
5*	OR271243	Laois	8 April 2022	nd	2 m
6	OR271244	Wexford	22 March 2022	Enteric symptoms, *Escherichia coli* co-infection	5 w
7	OR271245	Wexford	21 March 2022	Enteric and respiratory symptoms, *E. coli* co-infection	10 d
8	OR271246	Kilkenny	16 March 2022	nd	Calves (pooled)
9	OR271247	Kilkenny	14 March 2022	Pneumonia and enteritis, BHV-1 co-infection	4 w
10	OR271248	Cavan	23 February 2022	*Pasteurella multocida* and *M. haemolytica* co-infection	4 animals (pooled)
11	OR271249	Donegal	15 February 2022	Pneumonia	10 m
12†	OR271250	Wexford	11 March 2022	Pneumonia, other bacterial respiratory pathogens isolated	2 w
13†	OR271251	Wexford	11 March 2022	Pneumonia, other bacterial respiratory pathogens isolated	2 w
14	OR271252	Sligo	6 January 2022	nd	Weanling
15	OR271253	Laois	8 April 2022	nd	< 1 m
17	OR271254	Wicklow	23 May 2022	Pneumonia, BHV-4, *H. somni* and *M. haemolytica* co-infections	3 m
116	OR271255	Westmeath	2022	Dead	1 y male
117	OR271256	Offaly	2022	Pneumonia	8 m
119	OR271257	Meath	2022	Pneumonia	6 w
123	PP156983	Carlow	4 November 2022	Pneumonia, BRSV co-infection	9 m
124	PP156990	Kilkenny	3 November 2022	Respiratory symptoms	2 y female
125	PP156992	Kilkenny	6 December 2022	Respiratory and enteric symptoms, PIV-3 co-infection	8 m
126	PP156984	Cork	14 December 2022	Dead, respiratory and enteric symptoms, BRSV co-infection	6 m
127	PP156985	Galway	21 November 2022	Dead, pneumonia, BRSV co-infection	7 m
128	PP156986	Longford	2 December 2022	Respiratory symptoms, PIV-3/BRSV co-infections	5 w
129	PP156987	Carlow	26 October 2022	Respiratory and enteric symptoms, PIV-3 co-infection	5 m
131	PP156988	Wexford	10 November 2022	Pneumonia, enteric symptoms, BRSV co-infection	10 m
132	PP156991	Tipperary	6 January 2023	Enteric symptoms	10 m female
133	PP156989	Carlow	7 November 2022	Dead, pneumonia	8 m

BHV-1/4bovine alphaherpesvirus-1/4BRSVbovine respiratory syncytial virusddays oldmmonths oldnano animal informationndno disease informationPIV-3parainfluenza virus-3wweeks oldyyears old

### Recombination and selection analysis

BoCoV spike, HE, N, membrane and small envelope coding sequences were downloaded from the GenBank database (https://www.ncbi.nlm.nih.gov/). Identical and incomplete sequences were removed from datasets. Codon alignments for each gene were obtained using the Clustal Omega alignment algorithm [15,16] in Jalview version 2 [17,19]. Stop codons were deleted manually. Recombination breakpoints of each gene were predicted using Genetic Algorithm for Recombination Detection (GARD) [20] through the data monkey webserver (datamonkey.org) [21]. Sequences arising through recombination were removed from alignments and then mixed effects model of evolution (MEME) [22] and fast unconstrained bayesian approximation (FUBAR) [23] methods were used through the data monkey web server [21] to infer site-specific selective pressures. MEME predicted sites under diversifying episodic selection and FUBAR estimated synonymous and non-synonymous mutation rates to infer pervasive selective pressure.

### Mapping of immune epitopes

Immune epitopes for BoCoV structural proteins were mapped from previous studies. Firstly, BoCoV spike and HE major histocompatibility complex-I (MHC-I, also known as bovine leukocyte antigen-I) and B cell receptor (BCR) immune epitopes were predicted bioinformatically, although this manuscript has not yet been peer reviewed and is only available as a preprint [24]. The second two methods mapped immune epitopes identified for *Human betacoronavirus OC43* (HCoV OC43) protein onto BoCoV orthologues because, depending on the variants selected for comparison, spike, HE and N shared approximately 91%, 94 and 97% identity at amino acid level. Further, the two *Embecovirus* species are closely related, with evidence suggesting that HCoV OC43 evolved from BoCoV following a relatively recent zoonotic event [25]. MHC-II (also known as bovine leukocyte antigen-II) immune epitopes identified for HCoV OC43 by co-immunoprecipitation (co-IP) of MHC-II molecules [26] were mapped onto BoCoV spike, HE, nucleocapsid and small envelope. HCoV OC43 spike residues shown to interact with neutralising antibodies (nAb) through structural analysis or shown to be escape mutants following passage in the presence of antibodies [27] were also mapped onto BoCoV spike. These will be referred to as nAb-OC43 henceforth. Two neutralising antibody domains have been identified in BoCoV spike at positions 351–403 and 517–621 of the Mebus reference strain [28,30] with a single amino acid change in residue 528 resulting in loss of reactivity with some neutralising antibodies [31]. These will be referred to as nAb-BoCoV henceforth.

### Protein structures

BoCoV spike structure was predicted using SWISS-MODEL (https://swissmodel.expasy.org/) with the BoCoV Mebus spike amino acid sequence (GenBank accession number: AAA66399.1) as input [32]. BoCoV HE structure had been determined by X-ray crystallography [33] and deposited on the protein data bank (https://www.rcsb.org/). Spike and HE structures were visualised and annotated in PyMOL [34].

### Sequencing spike and haemagglutinin esterase

RNA from samples positive for BoCoV was converted to cDNA using the SuperScript III Reverse Transcriptase kit (Invitrogen) following the manufacturer’s protocol. cDNA was used as a template for PCR [Phusion HF kit (New England Biolabs)] with primers designed to amplify the spike and HE genes (Table 2). PCR products from reactions producing a specific fragment underwent PCR clean-up (Qiagen) and, if multiple fragments were detected, gel purification (Qiagen) from a 0.8% agarose gel was carried out. Purified fragments were sent for Sanger sequencing using appropriate forward and reverse primers (Eurofins Genomics).

**Table 2.** Primer pairs used to amplify regions of HE and spike genes. Start and finish positions of the amplicons are provided relative to the first nucleotide of the HE or spike genes. Primers were designed for this study or taken from [45,46]

Gene	Start	Finish	Primer sequences (5’–3’)		Annealing (°C)
			Forward	Reverse	
HE	−365	547	CCCTCATCACCGGCTAGACT	CCCCAAAATTAGCTTCACGAGC	65
HE	−288	476	CCACTGGATGGGAATTCGTTT	GTAGGTTGTGCAGAGCCATT	63
HE	377	1353	AGGCTTGTTTTACACTCAGGT	GTACACTTTAAATCTCCTATAACAGC	61
Spike	−107	1039	CTTGGCATTCTTTTGGGTGTTGC	TAATGGAGAGGGCACCGACTT	66
Spike	782	1550	GGGTTACACCTCTCACTTCT	GCAGGACAAGTGCCTATACC	60
Spike	1460	2286	GTCCGTGTAAATTGGATGGG	TGTAGAGTAATCCACACAGT	60
Spike	1855	2731	TTACAAAAATCAAACACAGACAT	AAACTTTATTACAATCGCTTCC	60
Spike	2680	3617	TCAATTTTTCCCCTGTATTAGG	GTAGTAATAACCACTACCAGTG	59
Spike	3475	4218	TTTAGCTATGTCCCTACTAAGTA	TGTGGTAGCTATTATAATATGCTCG	60

### Phylogenetic analysis

Reads obtained from Sanger sequencing were trimmed to remove low-quality base calls. Reads from the same sample were aligned to BoCoV spike and HE references using the Clustal Omega alignment algorithm [15,16] in Jalview [17,19]. References were selected based on the closest NCBI blast hits for individual sequences from the same sample. Sequences were deposited on GenBank (Table 1). Nucleic acid sequences were converted to amino acid sequences and then phylogenetic trees were generated using the neighbour-joining method [35] in Seaview version 5 [36]. Trees were annotated and images generated on the Interactive Tree of Life web server [37].

### Statistical analysis

Z scores were calculated to compare the proportion of codons under selection or occurrence of amino acid changes within or outside of functional domains and immune epitopes. Z score thresholds were calculated by determining the inverse of the Student’s *t*-test distribution using degrees of freedom [amino acid length of protein minus number of samples (1)] with a *P*-value cut-off of 0.05. When the Z score exceeded the threshold value there was a significant difference in proportions.

## Results

### Selection analysis of BoCoV structural proteins

**Fig. 1.**
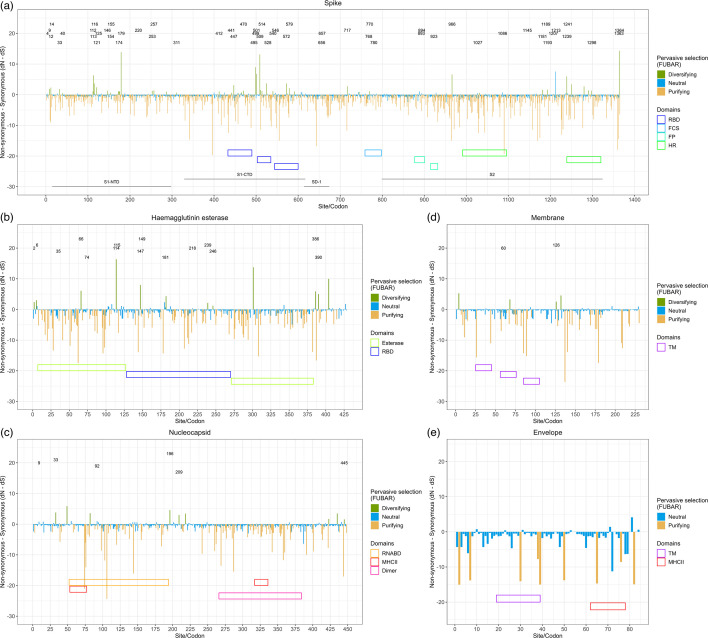
Selection analysis of structural BoCoV proteins. Bar plots show the results of selection analysis with FUBAR and annotated codons were found to be under diversifying selection using MEME for spike (**a**), HE (**b**), N (**c**), membrane (**d**) and small envelope (**e**) proteins. Locations of structural and functional domains were annotated at the bottom of plots. Dimer, interaction interface for dimerisation; FCS, furin cleavage site; FP, fusion peptide; HR, heptad repeat; RBD, receptor-binding domain; RNABD, RNA-binding domain; S1-CTD, S1 C-terminal domain; S1-NTD, S1 N-terminal domain; TM, transmembrane domain.

Regions of proteins under diversifying selection could represent immune epitopes, so having mapped several MHC-I, MHC-II, BCR and nAb epitopes onto BoCoV structural proteins, as described in the Methods section (Table 3), site-specific selective pressures were inferred using FUBAR and MEME algorithms (Fig. 1). Prior to running these, GARD analysis was used to identify and remove sequences derived from recombination. Recombinant sequences were detected in the spike gene alignment, but not genes of other BoCoV structural proteins. Spike (Fig. 1a) and HE (Fig. 1b) were under increased diversifying selection compared to N (Fig. 1c), membrane (Fig. 1d) and small envelope (Fig. 1e) proteins, as demonstrated by the increased proportion of codons under diversifying selection for spike and HE compared to other structural proteins (Table 4). An increased proportion of codons under purifying selection was observed for those encoding functional domains, but of all the functional domains annotated in Fig. 1, there was only significantly increased purifying selection within the N dimerisation motif. Receptor-binding domains (RBDs) are involved in protein–protein interactions and often contain nAb epitopes, so selection at spike and HE RBD was of particular interest. There was a significantly increased proportion of residues under diversifying selection within the RBD (*P*<0.01), but this was not the case for purifying selection (Fig. S1A, available in the online version of this article). For HE RBD there was an increased proportion of residues under diversifying selection, while there was a decreased proportion of residues under purifying selection, but these differences did not reach significance (Fig. S1B).

**Table 3.** Predicted immune epitopes and overlap with codons under diversifying selection and residues where amino acid changes were observed

Gene	Mapped immune epitope	Location		Diversifying selection (no. of codons)	No. of amino acid changes		Ref.
		Start	Finish		Compared to Mebus	Among Irish sequences	
Spike	MHC-I	11	19	2	1	0	[24]
		77	85	0	0	1	
		866	874	0	0	0	
		973	981	0	0	0	
		987	995	0	1	0	
		1004	1012	0	0	0	
		1302	1310	0	0	0	
	MHC-II	96	114	2	2	1	[26]
		267	292	0	0	1	
		770	785	1	2	0	
		911	930	2	1	0	
		1090	1113	0	1	0	
		1150	1165	0	0	0	
		1157	1174	0	0	0	
	BCR	721	727	0	0	1	[24]
		1205	1209	1	0	0	
	nAb-OC43	27	35	1	1	1	[27]
		81	–	0	0	0	
		242	–	0	0	1	
		253	258	2	3	1	
		267	–	0	0	0	
		357	367	0	0	0	
		395	402	0	0	0	
		413	417	0	0	0	
		458	459	1	1	1	
		461	–	0	0	0	
		463	–	0	0	0	
		483	–	0	0	0	
		490	502	4	2	2	
		531	533	1	2	1	
		536	557	2	1	2	
		582	583	0	0	0	
	nAb-BoCoV	351	403	0	0	3	[28,31]
		517	621	7	6	8	
HE	MHC-I	56	65	0	0	0	[24]
		186	194	0	0	0	
		360	368	0	2	2	
	MHC-II	93	108	0	0	1	[26]
		128	142	0	0	1	
		257	274	0	0	0	

**Table 4.** Selection analysis of structural proteins. Number and proportion of codons found to be under selective pressure for each BoCoV structural protein as determined by FUBAR and MEME

Protein	No. of codons under selection as determined by FUBAR and MEME (values in parentheses are percentage of codons in alignments under selection)						
	FUBAR (%)		MEME (%)	FUBAR+MEME	FUBAR only	MEME only	Diversifying total (%)
	Diversifying	Purifying					
Spike	49 (3.59)	570 (41.76)	57 (4.18)	33	16	24	73 (5.35)
HE	12 (2.80)	97 (22.66)	15 (3.50)	8	4	7	19 (4.44)
N	9 (2.00)	100 (22.27)	6 (1.34)	4	5	2	11 (2.45)
M	4 (1.74)	36 (15.65)	2 (0.87)	1	3	1	5 (2.17)
E	0 (0)	9 (10.71)	0 (0)	0	0	0	0 (0)

**Fig. 2.**
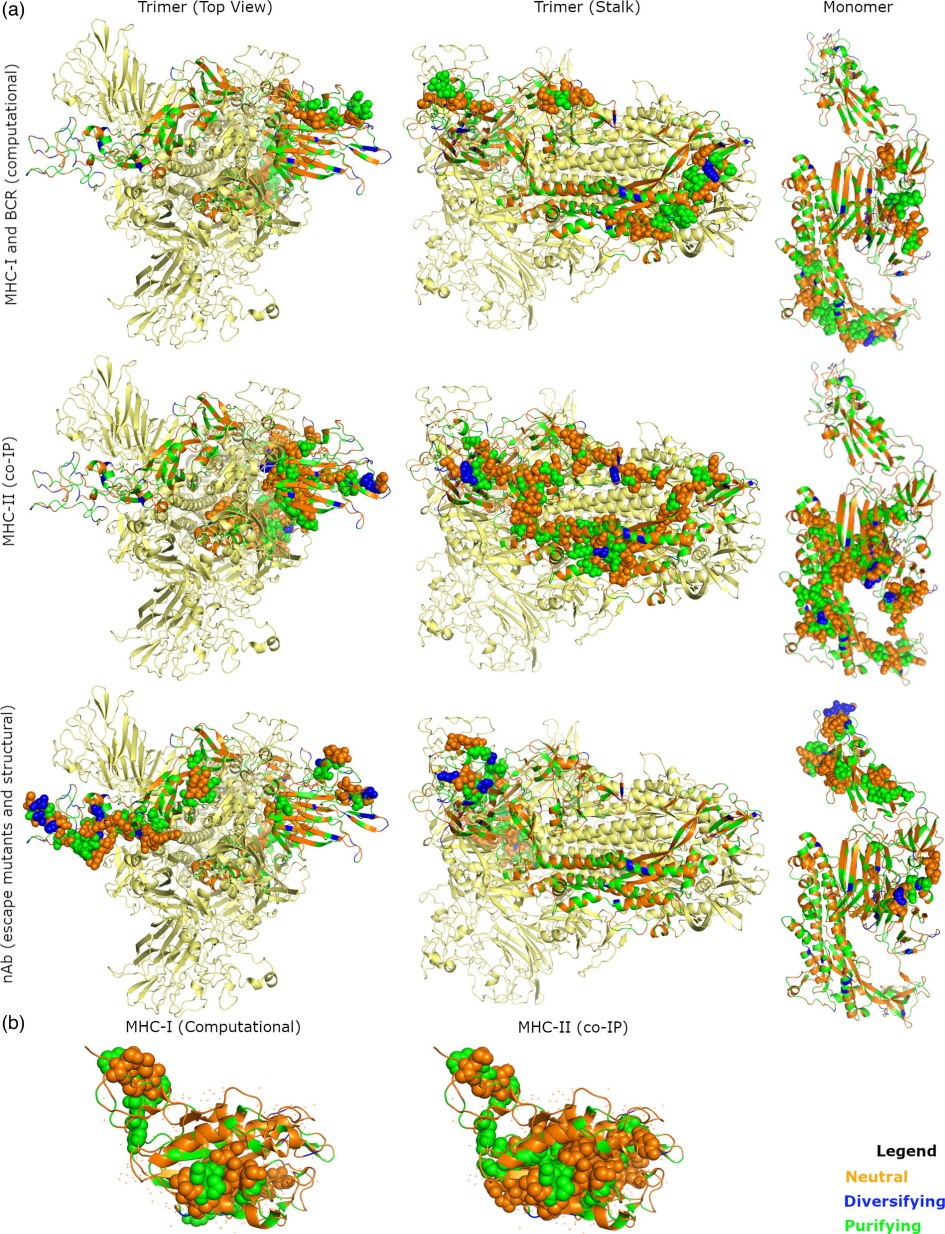
Spike trimer and HE structures with residues coloured based on results of FUBAR and MEME. Only one spike monomer was coloured. (**a**) Mapped spike immune epitopes predicted computationally, by co-IP of HCoV OC43 and by structural analysis of HCoV OC43 nAb–protein interactions are shown as spheres. (**b**) Mapped HE MHC-II and MHC-I immune epitopes shown as spheres. BCR, B cell receptor; co-IP, co-immunoprecipitation; MHC-I, major histocompatibility complex I; MHC-II, major histocompatibility complex II; nAb, neutralising antibody.

Amino acid changes occurring within immune epitopes can provide the virus with a selective advantage because this may reduce the effectiveness of the host’s immune response, so selection within mapped BCR, MHC-I, MHC-II and nAb immune epitopes was assessed (Table 3). Compared to codons not encoding immune epitopes, there was a significantly increased proportion of codons under diversifying selection for mapped immune epitopes in spike. When looking at the individual classes of immune epitopes, spike codons under diversifying selection overlapped one of seven spike MHC-I epitopes and one of two spike BCR epitopes predicted bioinformatically, but diversifying selection was not significantly increased in either epitope class (Fig. 2a). Coding sequences for three of seven spike MHC-II immune epitopes predicted by co-IP contained codons under diversifying selection, but diversifying selection was not significantly increased (Fig. 2a). Of the 87 residues predicted to contribute to nAb recognition (nAb-OC43), 12.64% were under diversifying selection, which was a significantly increased diversifying selection compared to the rest of the protein (Fig. 2a). Coding regions of the nAb-BoCoV domains also contained codons under diversifying selection but there was not significantly increased diversifying selection. Diversifying selection was not observed for any predicted HE MHC-I or MHC-II immune epitopes (Fig. 2b), or the two predicted nucleocapsid and one predicted small envelope MHC-II immune epitopes.

### Sequencing and phylogenetic analysis of BoCoV spike and HE

To compare currently circulating Irish isolates with older Irish isolates, international isolates and vaccine strains, the spike and HE genes of 29 BoCoV clinical isolates from cattle presenting with respiratory and sometimes enteric symptoms in Ireland were sequenced. Full-length spike and HE sequences were obtained for 27 and 22 isolates, respectively. Phylogenetic trees of Irish isolates obtained in this study were constructed with structural differences in the spike (Fig. S2A) and HE (Fig. S2B) trees indicative of recombination between BoCoV circulating in Ireland, which was supported by recombination breakpoint analysis of an HE–spike alignment (Fig. S3). One breakpoint was predicted in the HE gene at position 1240 and five were predicted in the spike gene at positions 769, 1272, 2365, 3322 and 3670. A phylogenetic tree of spike amino acid sequences from global isolates was constructed and some sequences from this study were in a subclade containing other Irish isolates collected in 2019/20, while most were in a subclade of French isolates from 2013/14 (Fig. S2C). A phylogenetic tree of global HE amino acid sequences showed that sequences from this study shared a subclade with Irish isolates collected in 2019/20, most formed a separate subclade with Albanian isolates collected in 2023, while sample 117 was in the Asian/American clade and closest to enteric Chinese isolates from 2018 and 2020 (Fig. S2D). The change in position of sample 117 for the spike and HE coding regions suggests that it has a hybrid sequence, which could be produced by recombination between a European and Asian/American isolate. This was confirmed by visual inspections of the alignment, which showed that sample 117 HE contains single-nucleotide variants more prevalent in Asian/American isolates between nucleotide positions 641 and 1084 (Table 5). Upstream of this region, the first European-specific single-nucleotide variant in sample 117 is in position 573, while downstream the first in is position 1137, suggesting that the recombination breakpoints giving rise to this hybrid sequence lie within this region. Recombination breakpoint analysis of the HE–spike alignment of all GenBank sequences and sequences from this study detected three recombination breakpoints, which all overlapped the spike gene at positions 247, 1771 and 3265 (Fig. S3). For sample 117, regions upstream of HE and downstream of spike both sat in the European clade.

**Table 5.** Region-specific single-nucleotide variants and the nucleotide observed in sample 117 and most commonly observed in Asian/American or European isolates

Position		641	709	715	993	1044	1084
Most common nucleotide	Sample 117	T	T	C	T	T	A
	Asian/American	T	T	C	T	T	A
	European	C	G	G	C	C	G

**Fig. 3.**
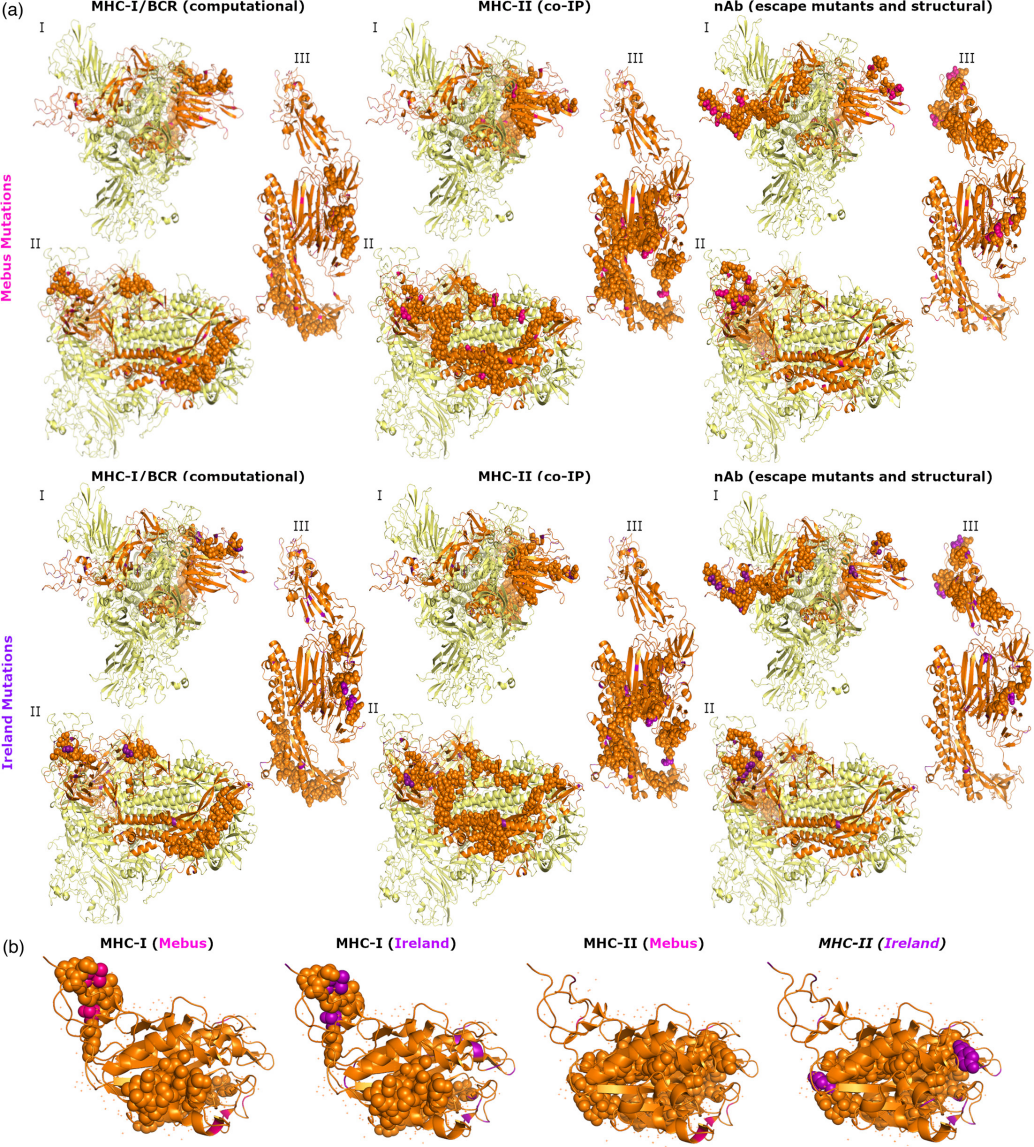
Overlap between residues with amino acid changes and mapped immune epitopes. (**a**) The spike trimer is presented as a cartoon, with predicted immune epitopes presented as spheres on one spike monomer in orange. Mebus mutations are shown in pink and Ireland mutations in purple. (**b**) Same as (a) but for HE.

Consensus sequences of spike and HE obtained in this study were translated and compared with BoCoV Mebus because this strain was used to design an inactivated vaccine often used in Ireland. Pressure to evade vaccine-mediated immunity would provide a selective pressure for amino acid changes occurring in immune epitopes of natural isolates so they diverge from vaccine strains. Compared to Mebus, there were amino acid changes in 48 (3.52%) spike residues and 9 (2.12%) HE residues of the Irish consensus sequences. As prior infection with natural isolates can generate an effective immune response against future infection, there could also be immune-driven selection for non-synonymous mutations among Irish BoCoV isolates. Among the spike sequences obtained in this study there were amino acid changes in 61 (4.47 %) residues, while in HE there were amino acid changes in 23 (5.42%) residues. Residues containing amino acid changes were grouped based on those different from Mebus (Mebus mutations) and those where variation was observed when comparing sequences from this study (Ireland mutations) (Fig. 3). Non-synonymous Mebus mutations overlapped 2 MHC-I epitopes (3.17%), 4 MHC-II epitopes (4.32%), 10/87 nAb-OC43 residues (11.49%) and 6/158 of nAb-BoCoV residues (3.80%) in spike (Fig. 3a). There was a significantly increased proportion of amino acid changes compared to Mebus in nAb-OC43, though this was not the case of nAb-BoCoV or any other class of immune epitope. Ireland mutations overlapped with 1 BCR (8.33%), 1 MHC-I epitope (1.59%), 2 MHC-II epitopes (1.44%), 9/87 nAb-OC43 residues (10.34%) and 11/158 nAb-BoCoV residues (6.96%) in spike (Fig. 3a). There was only a significantly increased occurrence of Ireland mutations in the nAb-BoCoV. One mapped HE MHC-I epitope overlapped Mebus and Ireland mutations (7.14%), while two mapped MHC-II epitopes overlapped Ireland mutations only (6.12%), but there was not a significantly increased occurrence of mutations in either epitopes class (Fig. 3b).

**Fig. 4.**
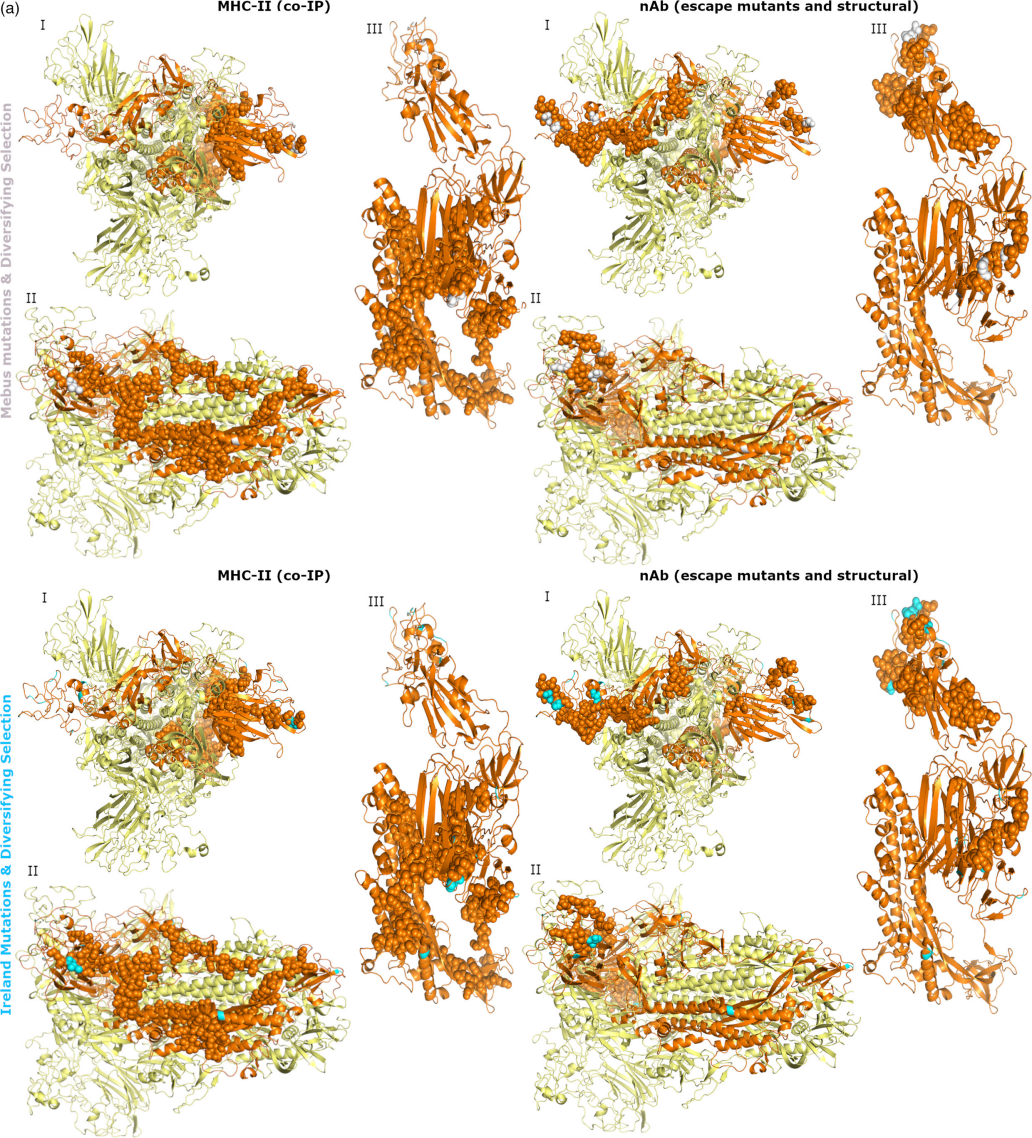
Overlap between residues where amino acid changes occurred and were under diversifying selection with mapped immune epitopes of spike. Mapped MHC-II (left images) and nAb-OC43 epitopes (right images) are shown as spheres and Mebus mutations under diversifying selection (top images) or Ireland mutations under diversifying selection (bottom images) are coloured white or cyan, respectively.

Combining the results of the selective pressure analysis and the positions of amino acid changes showed that 19/48 Mebus mutations and 20/61 Ireland mutations in spike were under diversifying selection. For HE, 2/9 Mebus mutations and 2/22 Ireland mutations were under diversifying selection. These residues did not overlap with any of the mapped HE immune epitopes or the mapped MHC-I and BCR immune epitopes of spike. Residue 113 of spike is a Mebus and Ireland mutation, under diversifying selection and overlaps with one mapped MHC-II epitope (96–114) (Fig. 4). Six out of eighty-seven nAb-OC43 residues were Mebus mutations and under diversifying selection (residues 33, 253, 257, 458, 499 and 501), while 5/87 nAb-OC43 residues were Ireland mutations and under diversifying selection (residues 257, 458, 499, 501 and 545) (Fig. 4). Two out of one hundred and fifty-eight nAb-BoCoV residues were Mebus mutations and under diversifying selection (residues 525 and 571), while 2/158 nAb-BoCoV residues were Ireland mutations and under diversifying selection (residues 525 and 545).

## Discussion

Previous studies were used to map immune epitopes onto structural BoCoV proteins. BoCoV spike and HE MHC-I and BCR immune epitopes were predicted bioinformatically [24]. HCoV OC43 spike, HE, N and envelope MHC-II epitopes were identified by co-IP [26] and then mapped onto BoCoV orthologues. Residues and domains involved in nAb recognition of HCoV OC43 spike were determined by structural analysis and identification of escape mutants [27] and then mapped onto BoCoV spike (nAb-OC43). Spike domains previously shown to be required for neutralisation by monoclonal antibodies [28,31] were also included in the study (nAb-BoCoV). Pressure to evade the immune response is one driver of diversifying selection in *Coronaviridae* [38,39], although there are other drivers of diversifying selection, such as adaptation to a new host [40]. Therefore, selective pressures acting on residues along BoCoV structural proteins were analysed to provide additional evidence as to the true nature of the mapped immune epitopes, with the assumption that some diversifying selection would be driven by immune escape. The proportion of codons under diversifying selection along the whole genes of spike and HE was increased compared to other structural proteins, suggesting that they are more likely to contain immune epitopes. There was no overlap between residues under diversifying selection and mapped HE immune epitopes, but there was significantly increased diversifying selection acting on codons of spike immune epitopes. A significantly increased proportion of nAb-OC43 codons were under diversifying selection, but this was not the case for other immune epitope classes. This suggests that pressure to evade the nAb immune response is driving BoCoV diversification.

There was also overlap between mapped immune epitopes and amino acid changes within the sequences from this study, suggesting that immune escape could be selecting for these changes. Once again, an increased proportion of amino acid changes was observed in nAb epitopes, implying that there is a selective advantage for non-synonymous mutations in regions recognised by nAbs. Selection for amino acid changes in predicted nAb epitopes suggests that nAbs are involved in an effective BoCoV immune response, which was further supported by diversifying selection acting on some of these residues. In the case of MHC-I, reduced support for these epitopes by selective pressure analysis could be due to downregulation of the MHC-I presentation by BoCoV. HCoV OC43 [41] and other *Coronaviridae* species have been shown to suppress MHC-I antigen presentation, which may reduce pressure for change at these locations compared to others [42,43].

Natural isolates in Ireland appear to be evolving away from the vaccine most widely used in Ireland because compared to the Mebus strain there were amino acid changes in 48 (3.52%) and 9 (2.12%) spike and HE residues of the Irish consensus sequences, respectively. There is evidence of amino acid changes occurring in regions potentially recognised by the immune response because 13 changes in spike and 4 changes in HE are within mapped immune epitopes. There were significantly increased Mebus mutations within nAb-OC43 epitopes, which could be caused by the immune response elicited by the Mebus-based vaccine selecting for non-synonymous mutations at these regions. Some residues had Mebus mutations, were under diversifying selection and overlapped mapped immune epitopes. It is currently unclear whether this evolution of the circulating BoCoV strains has reduced the effectiveness of the vaccine, but the rate of continual infection and disease associated with BoCoV suggests that it may be contributing. Designing a subunit vaccine based on currently circulating Irish clinical isolates of BoCoV could generate a more protective vaccine for cattle based in Ireland.

Almost all spike sequences from this study were in one of two clades, suggesting that most isolates descended from two distinct introductions of the virus into Ireland or diverged along two separate branches following a single introduction. Sample 129 formed an outgroup on its own and was the only isolate not grouped into the two main clades. This divergence from other sequences could be driven by adaptation to a unique environment or be the result of a third introduction of BoCoV into Ireland, which is less frequently detected. Less frequent detection could be caused by reduced sampling frequency associated with reduced pathogenicity, or it could be caused by reduced prevalence, which could indicate a recent introduction into Ireland. Another possibility is that this variant has a selective disadvantage, causing it to be outcompeted by other variants, reducing its prevalence. The relatedness of sample 129 to other isolates from this study was only based on the spike gene because full-length HE sequence was not obtainable. Most HE sequences from this study fell into one of two clades, which predominantly overlapped with the two main clades of the spike tree. Two isolates, namely sample 123 and sample 128, switched clade, suggesting that recombination had occurred around the 3′ end of the HE gene or the 5′ end of the spike gene, which was supported by GARD analysis showing breakpoints around both regions. Further sequence generation and analysis to support this was not possible due to a lack of material of sufficient quality. There was evidence suggesting that the HE coding region of sample 117 is a hybrid sequence resulting from recombination between an Asian/American isolate and European isolate. The presence of Asian/American-specific single-nucleotide variants between nucleotides 641 and 1084 suggests that the recombination event included at least this region, though this was not supported by GARD analysis. Compared to Asia and the Americas, HE sequences from European isolates on open-source databases are lacking, so more sequences are required to draw definitive conclusions on recombination events involving the HE gene. Such an event could have arisen due to mixing of cattle from Asia or the Americas with European cattle, enabling co-infection of an animal with European and Asian/American BoCoV isolates. Although national trading of cattle is common in Ireland, international movement of cattle is rare so such a mixing event is unlikely [44]. Alternatively, objects contaminated with BoCoV or non-bovine animals infected with BoCoV could have transported isolates between continents and facilitated the co-infection event giving rise to this recombination.

## Conclusion

This study mapped several immune epitopes onto BoCoV structural proteins based on previous research and several of those in spike and HE contain residues under diversifying selection or residues where amino acid changes have arisen in clinical isolates. Correlation with selective pressure and amino acid changes suggests that some mapped immune epitopes could be recognised by the bovine immune response. Overlap between predicted immune epitopes and amino acid changes observed in natural isolates compared to the Mebus vaccine strain means that immune evasion pressure could be one driver of diversification in BoCoV while reducing the protection afforded by Mebus-based vaccines. It is recommended that studies to assess the impact of this virus evolution on the effectiveness of currently used vaccines are undertaken to identify whether vaccines require updating. Further, phylogenetic analysis including sequences from this study showed a European isolate sat in the Asian/American clade, along with evidence of recombination between European and Asian/American isolates.

## supplementary material

10.1099/jgv.0.002019 Uncited Fig. S1.

## Abbreviations

BCR: B cell receptor
BoCoV: bovine coronavirus
FUBAR: fast unconstrained bayesian approximation
GARD: genetic algorithm for recombination detection
HE: haemagglutinin esterase
MEME: mixed effects model of evolution
MHC: major histocompatability complex
nAb: neutralising antibody
