# Supplementary material for: Diversifying selection identified in immune epitopes of bovine coronavirus isolates from Irish cattle
Source: J Gen Virol. 2024 Aug 19;105(8):002019. doi: 10.1099/jgv.0.002019 (PMC11333106; doi:10.1099/jgv.0.002019)
Supplement: Uncited Fig. S1. [file jgv-105-02019-s001.pdf]

Supplementary Information

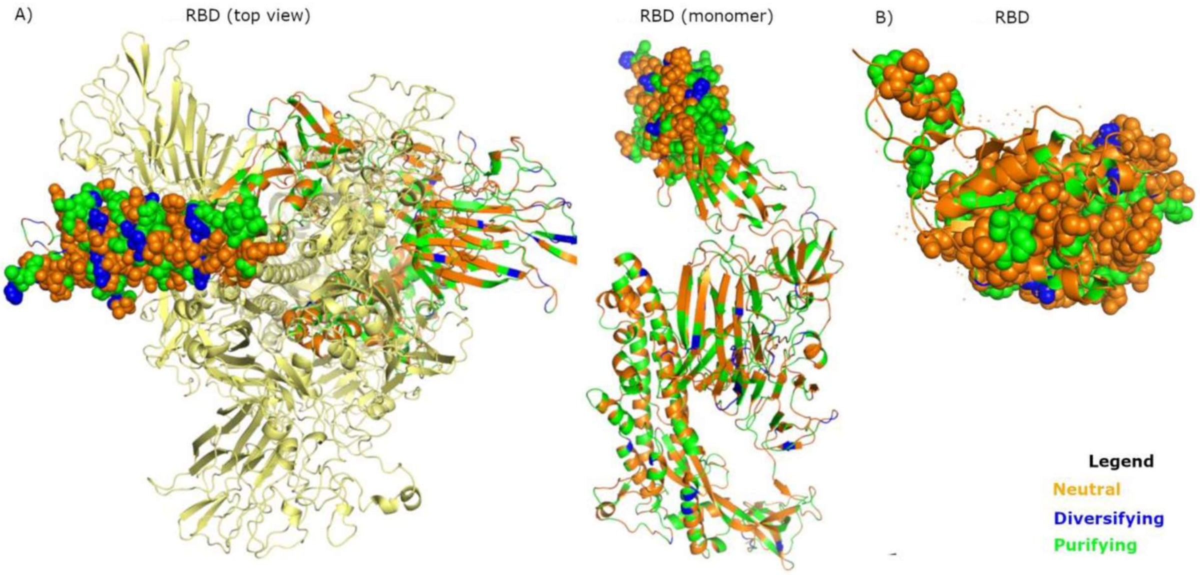

Supplementary Figure 1: RBD of spike trimer and HE monomer. Residues in the RBD of spike (A) and HE (B) are shown as spheres with residues coloured based on selection analysis of corresponding codons. Only one spike monomer is coloured.

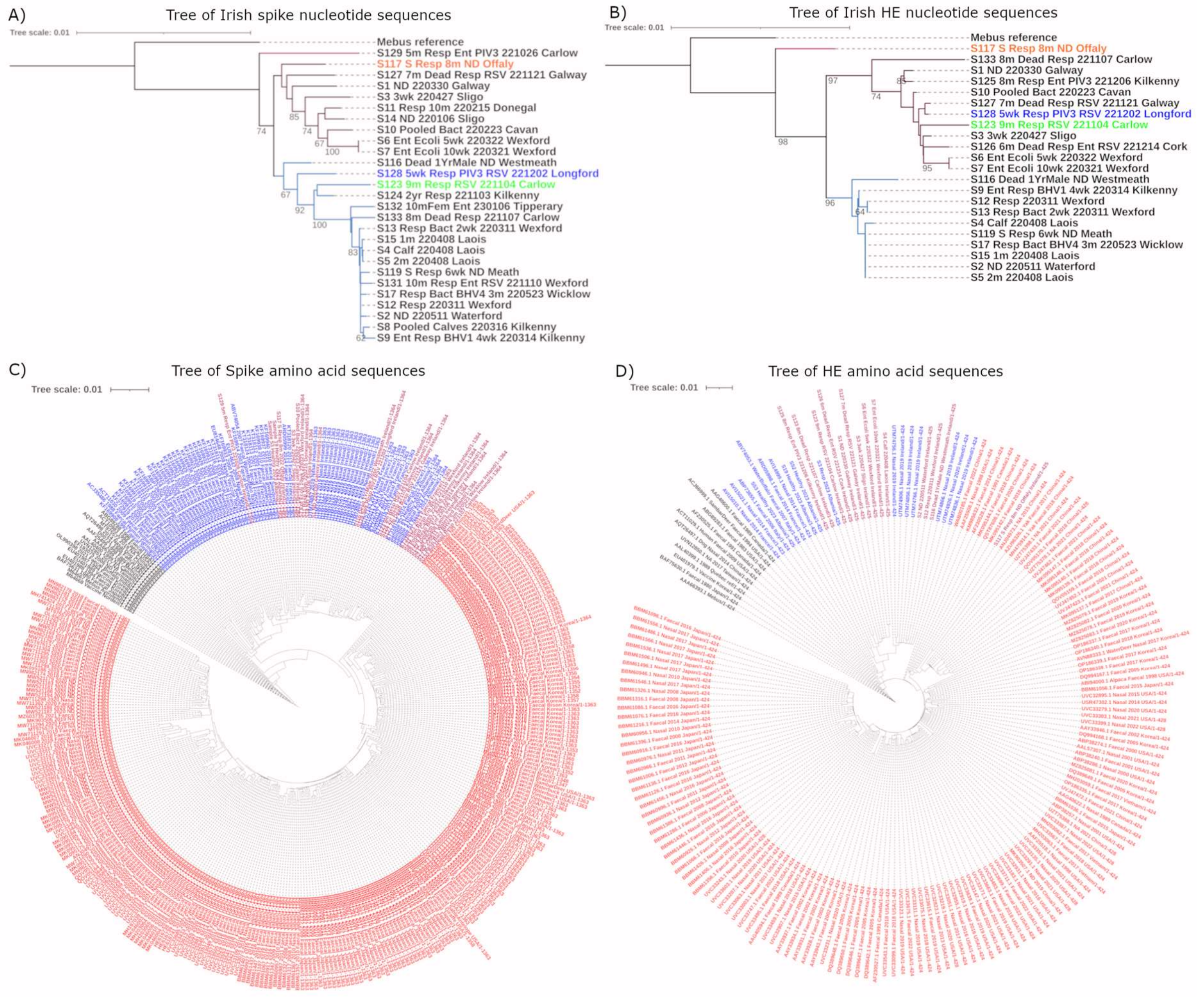

Supplementary Figure 2: Phylogenetic trees of BoCoV spike and HE generated using the neighbour joining method and rooted using the Mebus strain as an outgroup. A) Nucleotide sequences of spike for isolates from this study and the Mebus strain with different coloured branches indicating two main clades. B) Same as A), but for HE. The colours of branches for each clade match in A) and B), while isolates with coloured labels switched clades or formed their own outgroup. C) All unique spike amino acid sequences downloaded from GenBank, with label colours indicating location of isolation with Asian/American in red, European in blue and old isolates/vaccine strains in black. Sequences from this study are shown in pink. D) The same as C), but for HE.

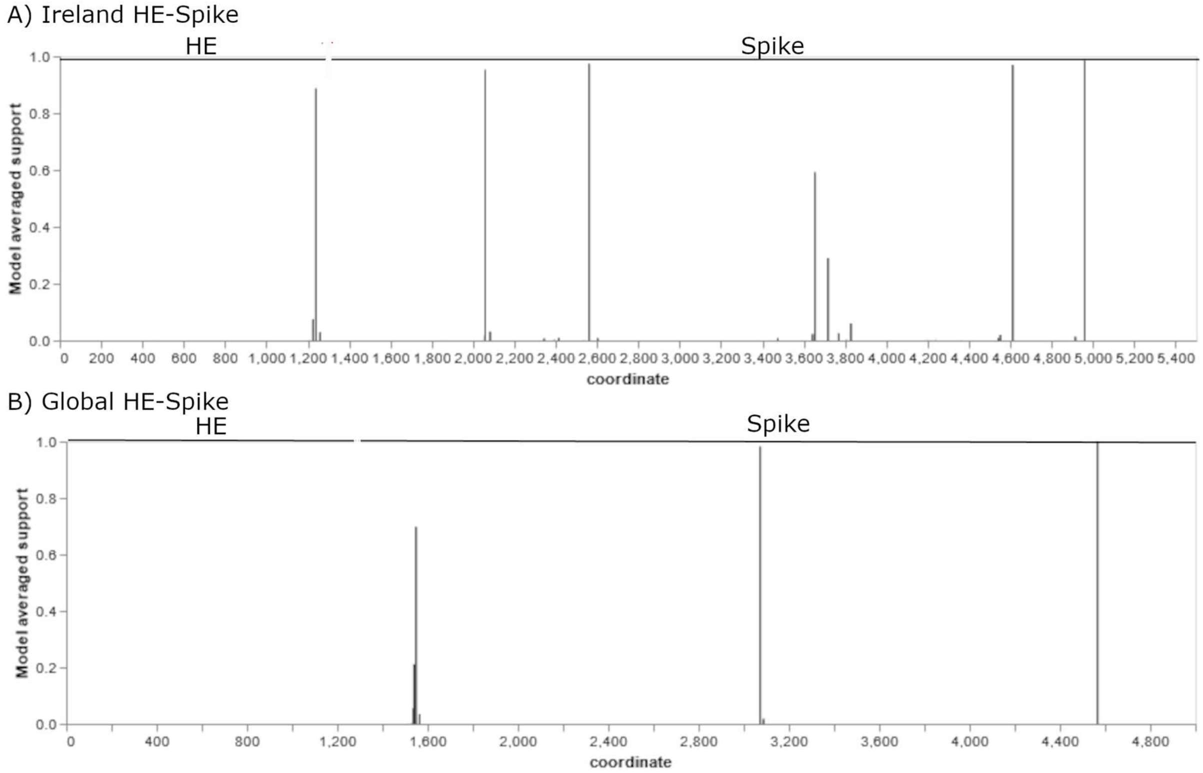

Supplementary Figure 3: Recombination breakpoints detected in HE-S nucleotide alignments by GARD. A) Sequences obtained in this study. B) GenBank sequences and sequences obtained in this study.
